# Glutathione-related substances maintain cardiomyocyte contractile function in hypoxic conditions

**DOI:** 10.1038/s41598-019-41266-2

**Published:** 2019-03-19

**Authors:** Yuri M. Poluektov, Irina Yu. Petrushanko, Nidas A. Undrovinas, Valentina A. Lakunina, Asker Y. Khapchaev, Valery I. Kapelko, Alexander A. Abramov, Vladimir L. Lakomkin, Mikhail S. Novikov, Vladimir P. Shirinsky, Vladimir A. Mitkevich, Alexander A. Makarov

**Affiliations:** 1 Engelhardt Institute of Molecular Biology, Russian Academy of Sciences, Vavilov St. 32, 119991 Moscow, Russia; 2 I.M. Sechenov First Moscow State Medical University, Ministry of Healthcare of the Russian Federation, Trubetskaya St. 8/2, 119991 Moscow, Russia; 3 National Medical Research Center for Cardiology, Ministry of Healthcare of the Russian Federation, 3rd Cherepkovskaya St. 15a, Moscow, 121552 Russia; 4 Department of Pharmaceutical & Toxicological Chemistry, Volgograd State Medical University, Pavshikh Bortsov Sq. 1, Volgograd, 400131 Russia

## Abstract

Severe hypoxia leads to decline in cardiac contractility and induces arrhythmic events in part due to oxidative damage to cardiomyocyte proteins including ion transporters. This results in compromised handling of Ca^2+^ ions that trigger heart contractile machinery. Here, we demonstrate that thiol-containing compounds such as N-acetylcysteine (NAC), glutathione ethyl ester (et-GSH), oxidized tetraethylglutathione (tet-GSSG), oxidized glutathione (GSSG) and S-nitrosoglutathione (GSNO) are capable of reducing negative effects of hypoxia on isolated rat cardiomyocytes. Preincubation of cardiomyocytes with 0.1 mM GSNO, 0.5 mM et-GSH, GSSG, tet-GSSG or with 10 mM NAC allows cells 5-times longer tolerate the hypoxic conditions and elicit regular Ca^2+^ transients in response to electric pacing. The shape of Ca^2+^ transients generated in the presence of GSNO, et-GSH and NAC was similar to that observed in normoxic control cardiomyocytes. The leader compound, GSNO, accelerated by 34% the recovery of normal contractile function of isolated rat heart subjected to ischemia-reperfusion. GSNO increased glutathionylation of Na,K-ATPase alpha-2 subunit, the principal ion-transporter of cardiac myocyte sarcolemma, which prevents irreversible oxidation of Na,K-ATPase and regulates its function to support normal Ca^2+^ ion handling in hypoxic cardiomyocytes. Altogether, GSNO appears effective cardioprotector in hypoxic conditions worth further studies toward its cardiovascular application.

## Introduction

Hypoxia of the myocardium is a frequent complication of a numerous pathological conditions, such as coronary heart disease, myocardial infarction, open heart surgery and preservation of an isolated heart. Intensive study of the problem over the past decades led to the discovery of several ways to reduce hypoxic effects on the myocardium. Among them, there is the use of cardioplegic solutions^1^ and ischemic preconditioning^2–4^. However, almost all these procedures are applicable only under special conditions and are of little use in the most frequent cardiac pathologies, such as ischemic heart disease and myocardial infarction.

In the first minutes of acute hypoxia/ischemia in the cell an increased formation of reactive oxygen species (ROS) begins, this leads to disruption of cellular metabolic processes, irreversible oxidation of proteins, and activation of membrane lipids peroxidation^5^. The increase of ROS levels leads to disruption of redox status of the cell and alters normal functioning of ion transporting systems. Oxidation and inhibition of ion transporters, in particular, Na,K-ATPase, is one of the first and critical events affecting the viability of cell^6^. Inhibition of Na,K-ATPase leads to disruption of the Na/K gradient and is often accompanied with the alterations of Na/Ca and Na/H exchangers that function in cooperation with the enzyme. Altogether, the disbalance in major ion gradients leads to elevation of intracellular calcium, a change of osmotic balance and ultimately to cell death.

Normal redox status of the cell is maintained by a number of enzymes, such as superoxide dismutase, catalase, glutathione peroxidase and low molecular weight antioxidants. The main component determining the redox status of cells is the tripeptide glutathione. The ratio of reduced to oxidized forms of glutathione (GSH/GSSG) is normally 100/1, and it decreases to 1/1 during oxidative stress^7^. Maintaining high levels of reduced glutathione (GSH) enhances antioxidant defense. GSH is involved in the neutralization of free radicals, being oxidized to GSSG. After that it can be reduced back to GSH by glutathione reductase. Additionally, covalent binding of glutathione protects thiol groups of intracellular proteins from irreversible oxidation by free radicals to sulfinic (-SO_2_H) and sulfonic (-SO_3_H) groups. After the induction of oxidative stress, an increase in the levels of GSSG occurs. GSSG interacts with -SH groups, while GSH interacts with partially oxidized –SOH groups, preventing their irreversible oxidation to -SO_2_H, -SO_3_H states. Following restoration of normal redox conditions in the cell, glutathione modifications are removed from proteins by the special enzymes, in particular, by glutaredoxin^7^. However, if the resources of the antioxidant defense system are not sufficient, irreversible oxidation of protein thiol groups leads to disruption of critical cellular functions^8^.

Glutathionylation of proteins belonging to ion transporting system leads to significant changes in their functioning, which is considered important for the adaptation of cells to hypoxia^6,7,9^. We have previously shown that acute hypoxia induces glutathionylation of Na,K-ATPase, which inhibits the enzyme^6,10^ and allows the cell to avoid depletion of ATP before switching to anaerobic glycolysis. We have found that the incubation of SC1 mouse fibroblasts with thiol-containing compounds, such as N-acetyl cysteine (NAC), the penetrating analog of GSH (et-GSH), oxidized glutathione (GSSG) and nitrosoglutathione (GSNO), induces an increase in glutathionylation of Na, K-ATPase, which results in an increase in cell viability under hypoxic conditions^11^. We hypothesized that the protective effect of the short-term ischemic preincubation is associated with glutathionylation of ion transporters that confers more effective antioxidant protection during long-term hypoxia. In addition, the thiol compounds could replenish the pool of glutathione, which can also have a positive effect.

Here, we demonstrate that several thiol-containing compounds including various glutathione derivatives significantly extend normal functioning of isolated rat cardiomyocytes under hypoxic conditions. The most effective of these derivatives, GSNO, was shown to increase glutathione incorporation in Na,K-ATPase of cardiomyocytes and to promote faster recovery of isolated rat heart contractility following ischemia-reperfusion.

## Results

### NAC, GSSG, et-GSH, tet-GSSG and GSNO support normal Ca^2+^ transients in cardiomyocytes during hypoxia

**Figure 1:**
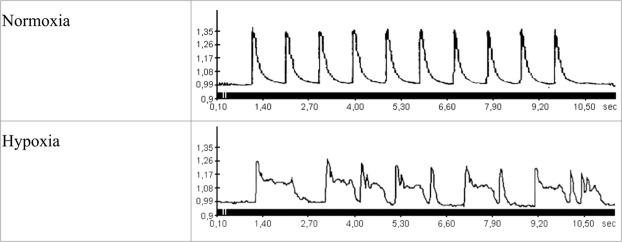
Changes in the concentration of free intracellular Ca^2+^ in isolated rat cardiomyocytes during electric stimulation (1 Hz) at normal oxygen level (buffer saturated with carbogen 95% O_2_ and 5% CO_2_) and under hypoxic conditions (buffer saturated with 95% N_2_ and 5% CO_2_). 10 sec fragments of the records, at 2 min of normoxia or hypoxia, are given. The ordinate shows the fluorescence intensity of Fluo-4 in arbitrary units (a.u.).

We used high-speed fluorescent microscopy to assess the dynamics of Ca^2+^ transients in isolated electrically stimulated cardiomyocytes under normal and hypoxic conditions (Fig. 1). In normoxia calcium peaks possess an asymmetric shape with sharp upward rise and more delayed downward descent. When cardiomyocytes are transferred to hypoxic solution the change in the shape of calcium peaks is readily observed. The descending arm of the peak broadens and acquires a dome-like appearance (Fig. 1), reflecting inefficient Ca^2+^ removal from the sarcoplasm. Ca^2+^ peaks loose regular appearance and hardly follow electric pacing rhythm. After 2–3 min of hypoxia cardiomyocytes stop responding to electric impulses by distinct Ca^2+^ peaks and perish soon.

**Figure 2:**
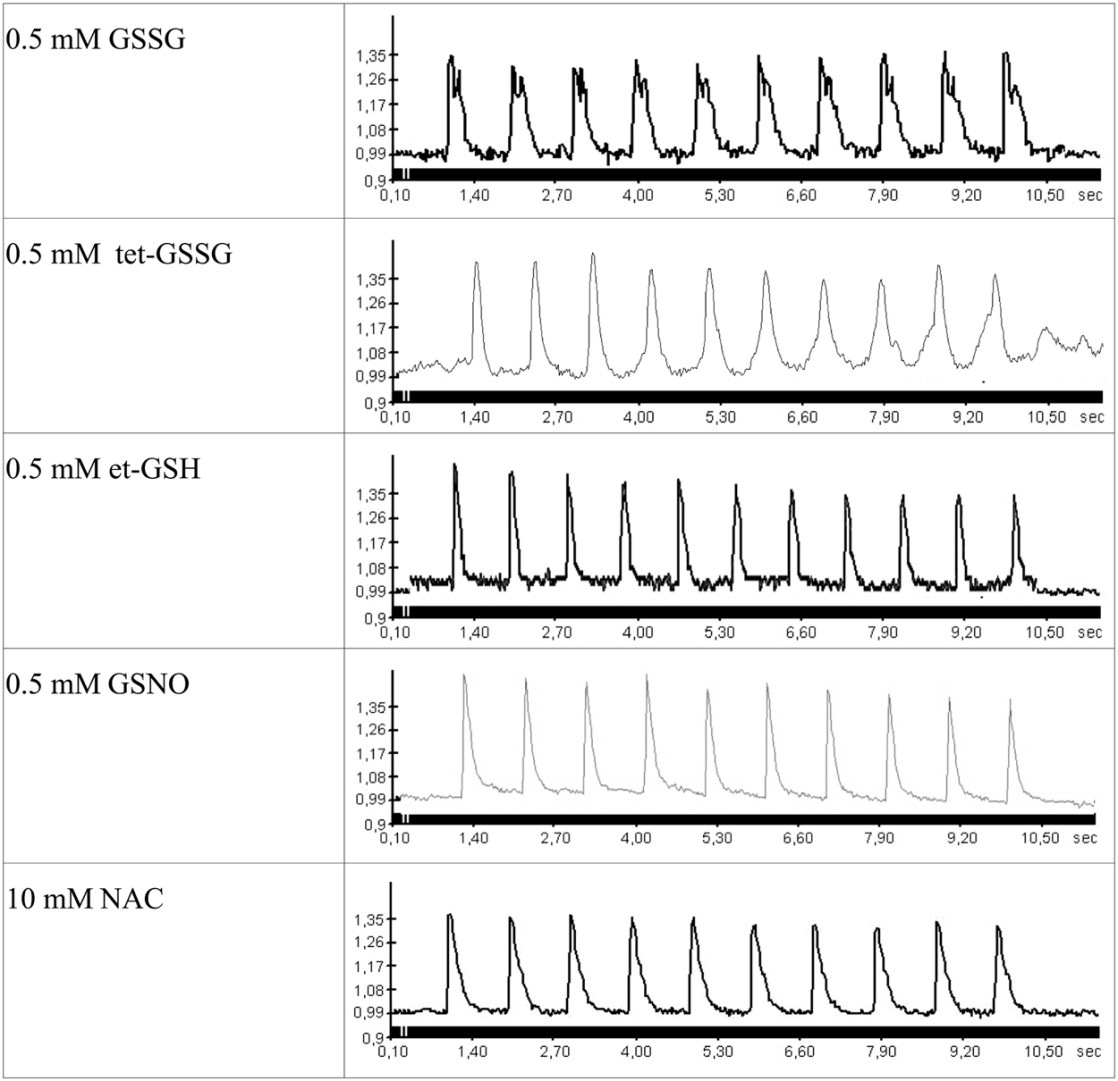
Changes in the concentration of free intracellular Ca^2+^ in isolated rat cardiomyocytes during electric stimulation (1 Hz) under hypoxic conditions (buffer saturated with 95% N_2_ and 5% CO_2_) in the presence of low molecular weight thiols. 10 sec fragments of the records in the presence of 0.5 mM et-GSH, 0.5 mM GSSG, 0.5 mM tet-GSSG, GSNO and 10 mM NAC, at 3 min of hypoxia are given. Similar recordings were obtained during the next 8–12 minutes of hypoxia depending on the substance added. The ordinate shows the fluorescence intensity of Fluo-4 (a.u.).

**Figure 3:**
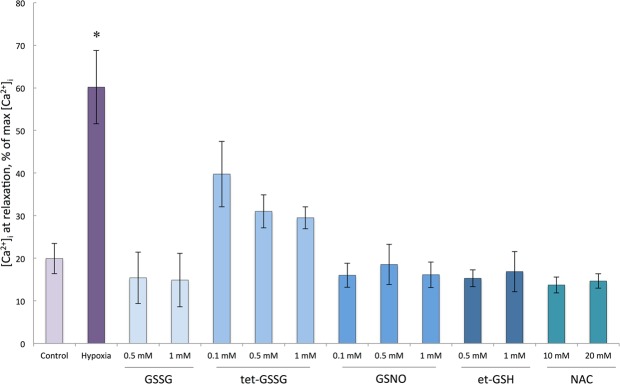
Relative levels of basal Ca^2+^ in rat cardiomyocytes in control, hypoxic conditions and in hypoxic conditions in the presence of et-GSH, GSSG, tet-GSSG, GSNO and NAC. The measurements were carried out at 2 min for control normoxia and hypoxia, and at 5 min for all other exposures. Average ratio ([Ca^2+^]_i_ at relaxation/[Ca^2+^]_i_ at peak) × 100% is presented for each condition. For all Ca^2+^ transients [Ca^2+^]_i_ at relaxation is determined at time point after Ca^2+^ peak in control normoxic cardiomyocytes when fluorescent signal reaches 20% of the peak value (on average at 250 msec). n = 11–17. Mean value ± S.D. Statistical analysis was performed using one-way ANOVA (*F* = 115.0, *p* < 0.00001) with post hoc testing (using paired samples Student’s t-test with Bonferroni correction); after a Bonferroni correction, a *p*-value < 0.003 was considered as statistically significant; *p < 0.001 for all conditions vs hypoxia.

**Figure 4:**
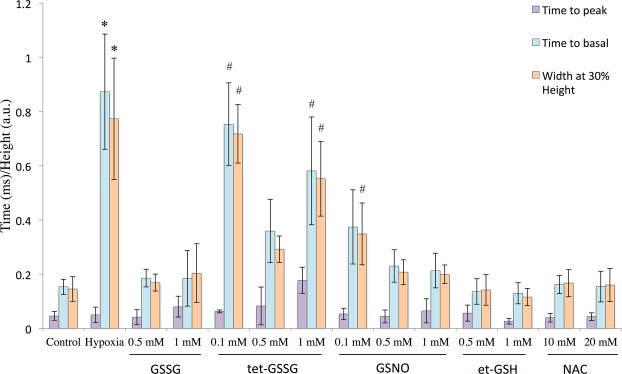
Calcium peak parameters (Time to peak, Time to basal and Width at 30% height) in control, hypoxic conditions and in hypoxic conditions in the presence of et-GSH, GSSG, tet-GSSG, GSNO and NAC. n = 18–25. Mean value ± S.D. Statistical analysis was performed using one-way ANOVA (for parameter «Time to peak» *F* = 3.4, *p* < 0.00099; for parameter «Time to basal» *F* = 24.4; *p* < 0.00001; «Width at 30% height» *F* = 23.5, *p* < 0.00001) with *post hoc* testing using paired samples Student’s *t*-test with Bonferroni correction. After a Bonferroni correction, a *p*-value < 0.003 was considered as statistically significant; *p < 0.003 for all conditions vs hypoxia excluding conditions marked #.

Preincubation of cardiomyocytes with et-GSH, GSSG, tet-GS, GSNO, and NAC for 20 min significantly increases their resistance to hypoxia. In the presence of these substances electrostimulated cardiomyocytes produce rhythmic and stable Ca^2+^ transients in the sarcoplasm over the period of 12–15 minutes, that is 4–5 times longer than in the absence of the substances (Figs 1–4). Cell maintained normal contractility rates all this time (Supplementary Figs 1, 2). We used the ratio of [Ca^2+^]_i_ at relaxation to [Ca^2+^]_i_ at peak as an integral parameter of bulk free sarcoplasmic Ca^2+^ dynamics over the period of ten seconds during which ten Ca^2+^ transients are elicited in response to electrostimulation in control cardiomyocytes. As shown in Fig. 3, all the substances except tet-GSSG maintained the value of this parameter comparable to that in normoxic cardiomyocytes and 3-fold lower than in hypoxic cells. The action of tet-GSSG on Ca^2+^ transients was less stable over the period of experiment and was accompanied by a gradual increase of basal Ca^2+^ toward the end of cell stimulation sequence that contributed to increased [Ca^2+^]_i_ relax/[Ca^2+^]_i_ max ratio. Next, we conducted a detailed analysis of Ca^2+^ transients. We found that the ascending arm of Ca^2+^ peak (Time to peak) is not significantly affected by either hypoxia or by the tested compounds (Fig. 4). On the opposite, the descending arm of Ca^2+^ peak (Time to basal) increases several-fold during hypoxia but is maintained at near control levels in the presence of the majority of the substances except of tet-GSSG (0.1 mM and 1 mM). Additional Ca^2+^ peak parameter (Width at 30% height) reflecting the duration of the transient also increases in control hypoxic cardiomyocytes as well as in cells treated with 0.1 mM and 1 mM tet-GSSG. Noteworthy, the behavior of this parameter closely followed (Time to basal) in all experimental conditions (Fig. 4).

Despite the fact that all substances tested significantly extend the period of rhythmic Ca^2+^ transients in hypoxic cardiomyocytes and the majority of compounds keeps basal sarcoplasmic Ca^2+^ at normoxic control levels, we observed differences in the shape of Ca^2+^ transients elicited in their presence (Figs 2, 4). The shape of control Ca^2+^ peaks is closely reproduced in the presence of 10 mM NAC. In the presence of 0.5 mM GSNO and 0.5 mM et-GSH Ca^2+^ the peaks become narrower in such a way that two subsequent Ca^2+^ transients may be separated by a “gap” of basal Ca^2+^. In the presence of 0.5 mM GSSG, Ca^2+^ peaks look bidentate apparently reflecting additional release of Ca^2+^ in the sarcoplasm.

Next, we determined the lowest effective concentration of the thiol-containing substances that extends rhythmic Ca^2+^ transients 5-fold or longer in hypoxic cardiomyocytes (Fig. 3). This concentration was: 10 mM for NAC; 0.5 mM for et-GSH, GSSG, and tet-GSSG; and 0.1 mM for GSNO. Additionally, we established that the protective effect of et-GSH and tet-GSSG could take place without preincubation due to the efficient penetration of these substances in cardiomyocytes. To implement the protective effect of GSSG, preincubation for 20 minutes is required (Supplementary Figs 3, 4).

### NAC, et-GSH, GSSG, tet-GSSG and GSNO have low acute toxicity in rat cardiomyocytes

All the thiol-containing substances used demonstrated low toxicity after 15 min incubation with isolated rat cardiomyocytes (Table 1). Cells maintained 80–100% viability when subjected to 10–100-fold excess of the substance sufficient to elicit protective effect in hypoxic cardiomyocytes. In particular, no acute toxicity was observed in the presence of 150 mM NAC and 10 mM GSNO.

**Table 1:** Percent of live cardiomyocytes after 15 min of incubation with et-GSH, GSSG, NAC, tet-GSSG, GSNO.

Compound	Concentration, mM	Viable cardiomyocytes, % (mean ± SD)
GSSG	0.5	98.4 ± 17.3
	10	92.0 ± 18.1
	50	90.6 ± 18.5
NAC	15	98.1 ± 19.8
	100	97.4 ± 20.5
	150	97.0 ± 21.3
tet-GSSG	0.5	97.3 ± 19.2
	10	81.3 ± 17.7
	50	80.2 ± 15.3
et-GSH	0.5	98.4 ± 16.3
	10	86.8 ± 18.1
	50	85.2 ± 18.7
GSNO	0.5	97.5 ± 20.0
	5	99.0 ± 14.1
	10	99.7 ± 19.5

The number of live cardiomyocytes in the control without an addition of substances was taken as 100%.

Based on obtained results, we selected GSNO as the most effective compound among tested glutathione-related substances capable of protecting isolated cardiomyocyte in hypoxic conditions and focused our further studies on this molecule.

### GSNO accelerates the recovery of rat heart contractility after ischemia/reperfusion

**Figure 5:**
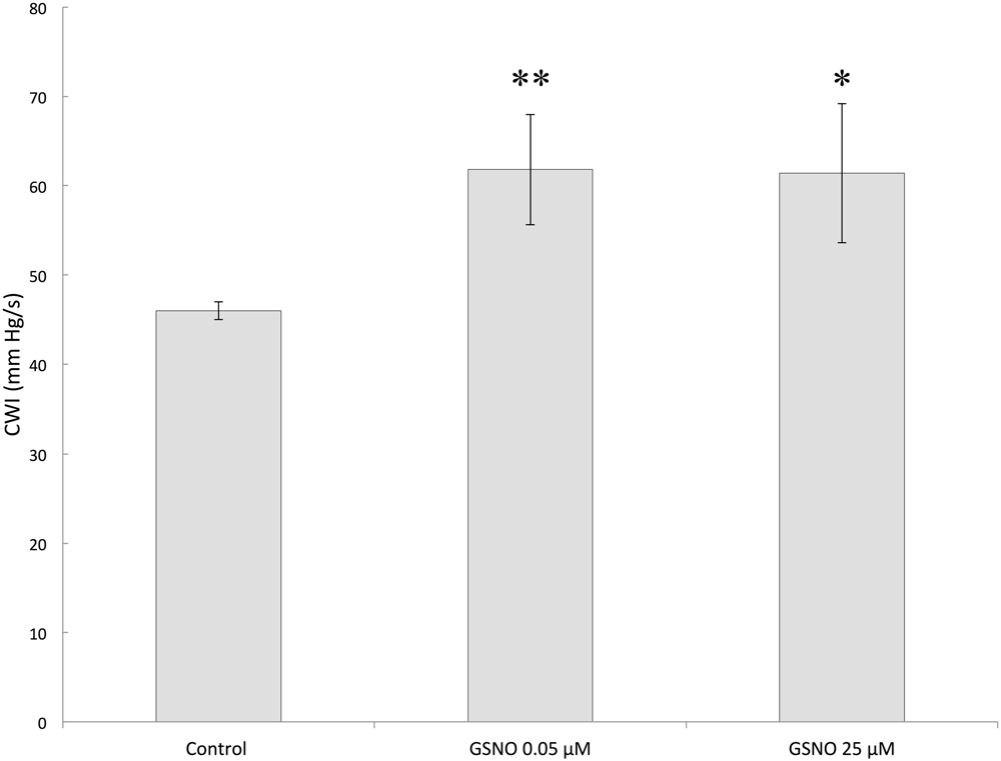
Restoration of the cardiac work index (CWI) after ischemia/reperfusion in the absence and in presence of GSNO n = 5, mean ± SD. Statistical analysis was performed using one-way ANOVA (*F* = 12.2, *p* = 0.00129) with *post hoc* testing (using paired samples Student’s *t*-test with Bonferroni correction); after a Bonferroni correction, a *p*-value < 0.016 was considered as statistically significant; *p < 0.003; **p < 0.0005.

The protective effect of GSNO was evaluated using the model of ischemia-reperfusion in isolated rat heart. We supplemented perfusing solution with 25 μM or 0.05 μM GSNO and delivered this substance to the heart as outlined in Materials and Methods. It was found that both concentrations of GSNO lead to faster recovery of the cardiac work index, CWI (Fig. 5). In the presence of 25 μM and 0.05 μM GSNO, the restoration of normal contractile function of the heart increased by 33% and 34%, respectively, compared to the control in the absence of GSNO (Fig. 5).

### GSNO stimulates glutathionylation of tissue specific α2 isoform of Na,K-ATPase in rat cardiomyocytes

**Figure 6:**
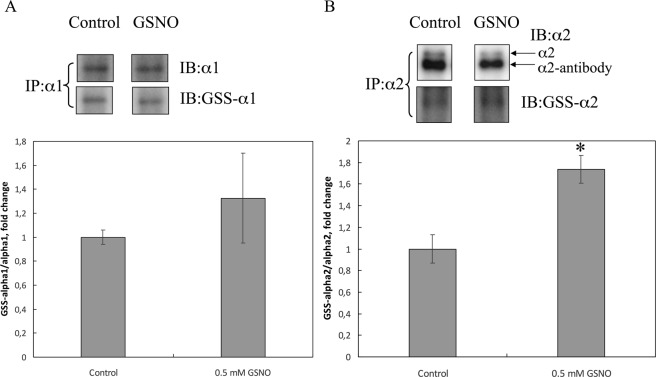
S-glutathionylation of α1 and α2 isoforms of Na,K-ATPase catalytic-subunit after GSNO treatment of cardiomyocytes. α1-Subunit (**A**) or α2-subunit (**B**) of Na,K-ATPase was immunoprecipitated (IP) from cell lysates by anti-α1 antibodies or anti-α2 antibodies and glutathionylation was detected with anti-glutathione (anti-GS) antibodies. The original immunoblotting readouts are presented above. Bars represent changes in the S-glutathionylated (GSS- α1/α1) or (GSS- α2/α2) form of the protein normalized to its total amount; n = 3, mean ± SD; *p < 0.05. Full-length blots are included in Supplementary materials (Supplementary Fig. 7).

In order to identify the level of glutathionylation of ubiquitous α1 and tissue specific α2 subunit of Na,K-ATPase under the influence of GSNO, we made immunoprecipitation of these proteins followed by western blot analysis. The results are shown in the Fig. 6. According to our data GSNO leads to an increase in glutathionylation of tissue specific α2 subunit of Na,K-ATPase, but the glutathionylation of the α1 isoform does not change significantly. These data are in good agreement with fact that α2 isoform of Na,K-ATPase is more sensitive to glutathionylation than α1 isoform^6^.

## Discussion

**Figure 7:**
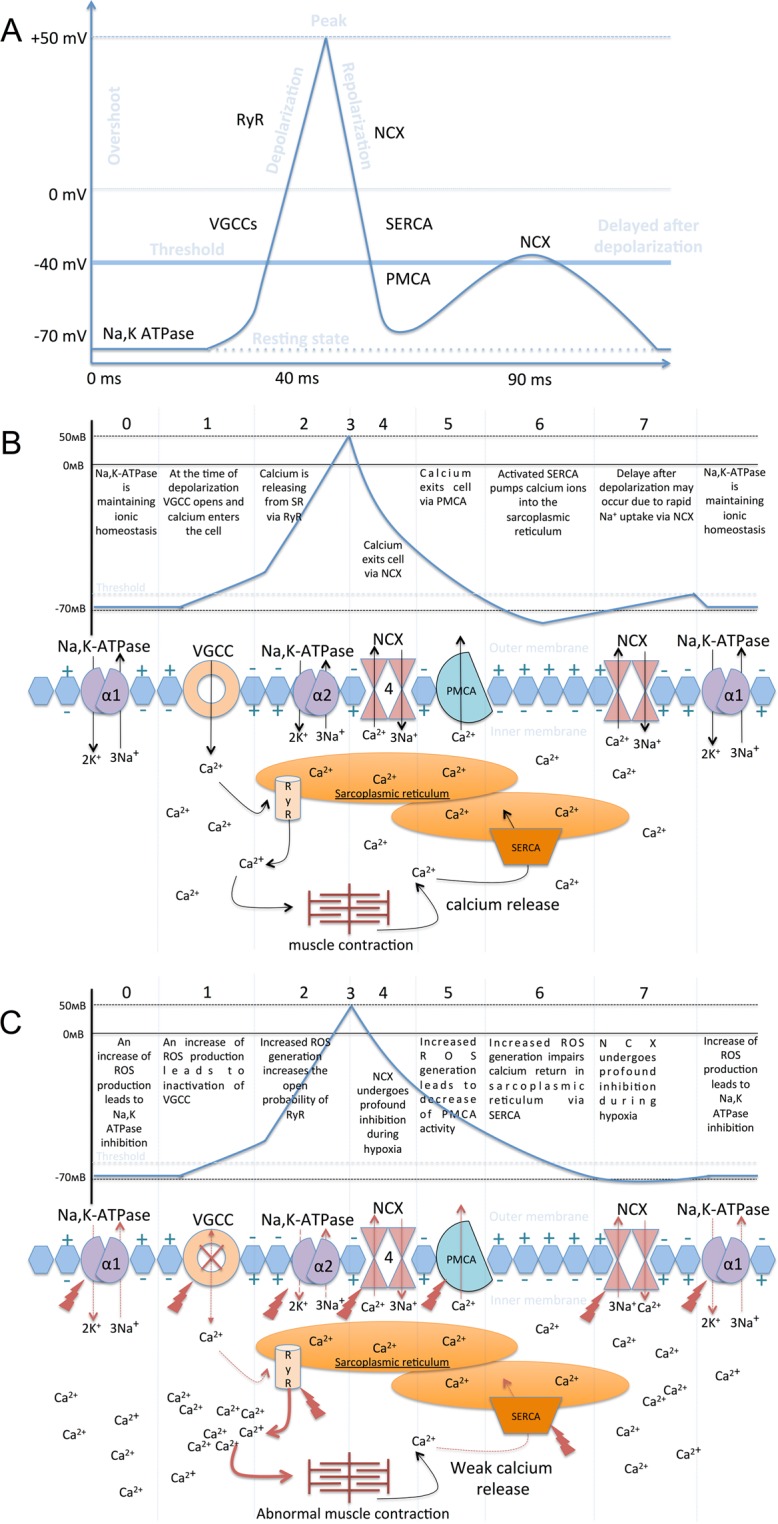
Scheme depicting activation of ion-transporting enzymes in cardiomyocytes during electrochemical coupling and actomyosin contraction. (**А**) The projection of activation of ion-transporting enzymes on the action potential of cardiomyocytes. (**B**) Activation of ion-transporting enzymes during the action potential of cardiomyocytes. At rest, the membrane charge is maintained by Na,K-ATPase and Na/Ca exchanger (NCX) enzymes. During stimulation, potential-dependent membrane calcium channels (VGCC) become permeable, calcium enters the cell and activates the ryanodine receptors (RyR2) of the sarcoplasmic reticulum (SR). Calcium released from SR interacts with troponin-myosin complex, which leads to muscle contraction. Calcium released from troponin-myosin complex back to the cytosol activates the Ca-ATPase of the sarcoplasmic reticulum (SERCA2), which begins to pump Ca^2+^ back into the SR, in parallel Ca^2 +^ is pumped out of the cell via membrane calcium ATPase (PMCA) and NCX. (**C**) Effect of acute hypoxia on ion-transporting enzymes. Increased ROS production, which occurs soon after the onset of hypoxia, leads to Na,K-ATPase inhibition. Tissue specific α2-subunit Na,K-ATPase which is important for regulating of Ca^2+^ levels is more redox sensitive than ubiquitous α1- subunit. So disturbance of activity of α2-containing isozyme is one of the first events during hypoxia that affect calcium transients. Inhibition of both isoforms of Na,K-ATPase results in Ca^2+^ overload, because the increased level of intracellular Na^+^ reverses NCX activity. Also, oxidative damage of VGCC leads to decline of their permeability and disruption of the Ca^2+^ uptake. ROS increases the open probability of RyR, which leads to excessive Ca^2+^ efflux into the cytosol from sarcoplasmic reticulum. PMCA and SERCA are also redox sensitive enzymes, and hypoxia leads to their inhibition, thus Ca^2+^ can not be driven out of the cell.

Under conditions of hypoxia, myocardial contractility worsens partially due to disorganized work of cardiomyocyte ion transporters. The levels of free Ca^2+^ ions in cardiomyocyte sarcoplasm fluctuate periodically because of highly coordinated activity of various type ion channels, transporters and exchangers (Fig. 7). The influx of external Ca^2+^ in cardiomyocytes results from the opening of voltage gated calcium channels (VGCC) that respond to action potential and sarcolemma depolarization. Shortly after that, the activation of ryanodine receptors (RyR2) releases Ca^2+^ from the sarcoplasmic reticulum leading to the net increase of Ca^2+^ in sarcoplasm and triggering actomyosin contraction. As a negative feedback, elevated Ca^2+^ signals to Ca^2+^ sequestration system members such as Na/Ca exchanger (NCX), Ca-ATPase of the sarcoplasmic reticulum (SERCA2) and plasma membrane calcium ATPase (PMCA) that promptly remove Ca^2+^ from sarcoplasm and stop actomyosin interaction^12^ (Fig. 7).

Unlike Ca^2+^ influx, that is gradient-driven and energy-independent Ca^2+^ removal from sarcoplasm requires ATP to support SERCA2 and PMCA as well as Na,K-ATPase, the principal ion transporter of cardiomyocytes and other cells. Besides maintaining Na/K gradient across plasma membrane and keeping resting potential Na,K-ATPase supports the direct mode of NCX ensuring that the exchanger expels one Ca^2+^ ion from cardiomyocyte bringing in three Na^+^ ions^13^. Na,K-ATPase promptly removes these sodium ions outside and reestablishes the gradient. Inhibition of Na,K-ATPase and accumulation of Na^+^ in sarcoplasm reverses NCX and it starts to expel Na^+^ and import Ca^2+^. Additionally, SERCA2 and PMCA are regulated by a specialized set of proteins that control Ca^2+^ pumping efficiency of these transporters^14,15^. Overall, Ca^2+^ extrusion system of cardiomyocytes appears more complex than Ca^2+^ influx system and, therefore, more prone to damage. It is currently viewed by many researches as an initiator of pathological heart contractility. The findings presented in this report are consistent with this view. The downward arm of Ca^2+^ transients is distorted and enlarged in hypoxic cardiomyocytes indicating the deficiency of Ca^2+^ extrusion system whereas Ca^2+^ influx system seems relatively intact.

Several possible molecular mechanisms could account for these alterations. It is well documented that the functioning of ion transporters depends on redox conditions in cells^6,13,16^. The onset of hypoxia leads to a change in intracellular redox status, increased production of reactive oxygen species^5^ and consequent oxidative damage to proteins. Under conditions of moderate hypoxia, the cell uses glutathionylation to prevent irreversible oxidation of proteins and to regulate their activity. An increase of the level of GSSG and depletion of NO in cardiomyocytes during hypoxia create favorable conditions for protein glutathionylation. In particular, we have shown that perfusion of the heart with hypoxic blood for 30 min leads to a decrease of Na,K-ATPase activity in myocardial tissue due to increased glutathionylation^6^ and reduced nitrosylation of its α-subunit^6,16^. We hypothesized that substances inducing glutathionylation of proteins contribute to the preservation of the functional activity of ion transporters and the contractile activity of cardiomyocytes under conditions of hypoxia.

To explore this possibility, we used thiol-containing substances that could increase glutathionylation of Na,K-ATPase^10,11^. NAC is a cysteine analog that penetrates into the cell and promotes the synthesis of glutathione^17^. et-GSH is a penetrating glutathione analog that is de-esterified in the cell to form glutathione, restoring the antioxidant properties of the cell^18^. GSSG is a dimer of glutathione, the content of which in cells increases during oxidative stress and hypoxia. Unlike other studied substances, GSSG is unable to penetrate the cells. In order to investigate the effect of oxidized glutathione, we synthesized cell-penetrating analog of glutathione - tet-GSSG. Cell treatment with this substance creates the most favorable conditions for glutathionylation of intracellular proteins, without returning the redox potential to the normal state. The synthesis of GSNO increases in cells in pathologic conditions, such as ischemia-reperfusion. GSNO can nitrosylate or glutathionylate protein thiols, or decompose to form a regulatory NO molecule that can penetrate cells. In addition, extracellular GSNO forms S-nitroso-L-cysteine, which penetrates cells through the L-system of amino acid transport. In the cytoplasm it nitrosylates proteins or GSH with the formation of intracellular GSNO^19^.

We found that all these compounds restore rhythmic Ca^2+^ transients in electrically paced cardiomyocytes subjected to hypoxia. This is achieved mainly through the maintenance of [Ca^2+^]_i_ sequestration/extrusion activity of cardiomyocytes, which decreases quickly and profoundly during hypoxic state. Interestingly, [Ca^2+^]_i_ mobilization activity of cardiomyocytes was not affected in our experimental model of hypoxia. Thus, ion-transporting systems of cardiomyocytes responsible for Ca^2+^ cycling in the sarcoplasm are prone to hypoxic inactivation while thiol-containing compounds target these systems and protect their functionality. Neither of the tested substances were toxic to cardiomyocytes when used in minimal effective concentration as well as in 10–100 excess over it indicating the potential for further medical use. However, substance-specific effects on Ca^2+^ transients allowed select the leader. Concentration wise, the most effective substance was GSNO that exerted protective action at 0.1 mM, еt-GSH had similar effect at 0.5 mM; the least effective was NAC acting at 10 mM. tet-GSSG demonstrated narrow therapeutic window whereas plasma membrane impermeant GSSG required preincubation to exert protective effect. Additionally, the latter substance induced bidentate Ca^2+^ peaks not typical for electrically paced intact cardiomyocytes. Based on comparative assessment, we selected GSNO as the most promising thiol compound for further studies.

Along these lines, we demonstrated that GSNO increases glutathionylation of Na,K-ATPase α2-subunit in cardiomyocytes and accelerates the recovery of normal contractility of isolated perfused rat heart after ischemia-reperfusion. These two activities of GSNO could be related. It is known that glutathionylation of regulatory cysteine residues in Na,K-ATPase leads to its inhibition^6^, elevated sarcoplasmic Ca^2+^ due to NCX working in reverse mode and transient increase in myocardial contractility due to earlier Ca^2+^ -induced Ca^2+^ -release (CICR) from the sarcoplasmic reticulum during excitation-contraction coupling^20,21^. However, excessive inhibition of Na,K-ATPase leads to Ca^2+^ overload and spontaneous Ca^2+^ release from the sarcoplasmic reticulum that can trigger cardiac arrhythmias^21^.

It was found that tissue-specific α2 subunit of Na, K-ATPase is functionally more important for regulating transient Ca^2+^ levels and contraction of cardiac muscle than the restorative α1 subunit, which carries out more generalized control over Na^+^ and K^+^ transport^21–23^. Na,K-ATPase α2 isoform is also more sensitive to glutathionylation than α1 isoform^6^ and according to our data GSNO induces glutathionylation α2 isoform, but not of α1 isoform (Fig. 6). It is proposed that specific inhibitors of α2 will be able to induce positive inotropic effect without triggering Ca^2+^ overload and arrhythmias^21^. Our findings suggest that GSNO could work as α2 subunit inhibitor in cardiomyocytes. Whether it is mediated by direct GSNO-dependent glutationylation of α2 subunit or through more complex modifying circuits remains to be elucidated. Submicromolar concentrations of GSNO that exert protective effect in the heart strongly (Fig. 5) suggest that NO-dependent signaling mechanisms are implicated in this process.

Incubation of cardiomyocytes under conditions of hypoxia leads to an increase in glutathionylation and a decrease in the activity of Na,K-ATPase, which correlates well with the data obtained on an isolated heart^5,16^. Apparently, this is one mechanism of cell adaptation to short-term hypoxia. Preincubation of cells with glutathione-related compounds also induces glutathionylation (Fig. 6) that can lead to a decrease in enzyme activity, which allows the cell to save ATP under conditions of hypoxia, prolongs the time of normal contractility of cardiomyocytes and their lifetime under hypoxia. Increased ROS production, which occurs soon after the onset of hypoxia, leads to Na,K-ATPase inhibition. Tissue specific α2- subunit of Na,K-ATPase which is important for regulating of Ca^2+^ levels is more redox sensitive than ubiquitous α1- subunit. So disturbance of activity of α2-containing isozyme is one of the first events during hypoxia that affect calcium transients. Inhibition of both isoforms of Na,K-ATPase results in Ca^2+^ overload, because the increased level of intracellular Na^+^ reverses NCX activity. Also, oxidative damage to VGCC leads to decline of their permeability, thus the Ca^2+^ uptake via VGCC is interrupted. ROS increases the open probability of RyR, which leads to the excessive Ca^2+^ efflux into the cytosol from sarcoplasmic reticulum. PMCA and SERCA are also redox sensitive enzymes, and hypoxia leads to their inhibition, thus Ca^2+^ cannot be driven out of the cell. Altogether after the onset of hypoxia severe calcium overload occurs which results in abnormal muscle contraction and widening of calcium peaks (Fig. 7).

Preincubation of cells with glutathione-related compounds also induces glutathionylation of Na,K-ATPase α2- subunit which leads to partial enzyme inhibition, and induces positive inotropic effect without triggering Ca^2+^ overload. Glutathionylation of VGCC can increase their permeability and decreases permeability of RyRs, which results in coordinated calcium influx from extracellular space and from SR. PMCA and SERCA also undergo glutathionylation and it leads to timely calcium efflux. Thus glutathionylation can promote of work coordination of ion-transporting systems under hypoxic conditions.

However, the mechanism of action of GSNO, apparently, is more complicated. Since this substance is capable of inducing not only glutathionylation inhibiting the Na,K-ATPase activity, but also nitrosylation causing its activation^16^, its effect on the activity of α2 and α1 subunits of Na,K-ATPase, having different sensitivity to redox modifications can be different and, probably depends on intracellular conditions.

It could not be excluded that thiol-containing substances used in this study exert their protective action on cardiomyocytes through multiple mechanisms. For instance, et-GSH, and NAC, similarly to GSNO, can directly deactivate ROS and interact with -SOH groups of proteins, protecting them from irreversible oxidation. GSSG and tet-GSSG are not capable of neutralizing ROS and their protective function is realized mainly through glutathionylation of thiol groups of proteins. Since the restoration of normal contractility of cardiomyocytes occurs in all cases, the determining factor of the protective effect of the investigated substances seems to be the glutathionylation of transport proteins.

SERCA2 activity increases following glutathionylation and nitrosylation, which improves calcium removal from sarcopalsm and protects the enzyme from inactivation by ROS^13,24^. Inhibition of NCX in hypoxia and its activation during reoxygenation was observed in guinea pig cardiomyocytes^25^, however, NCX glutathionylation was not described. PMCA can also be inhibited by hypoxia^26^, and in this state contribute to delayed calcium efflux. However, the role of PMCA is considered less significant in cardiac relaxation than the role of SERCA2 and NCX^13^.

RyR2 are sensitive to the changes in the redox status of the cell and could be inhibited at high and activated at low GSH/GSSG ratio. Nitrosylation of RyR2 by nitrosothiols, including GSNO, leads to channel activation^13,27^ while selective prevention of S-nitrosylation and S-glutathionylation of RyR2 is associated with higher instances of arrhythmia and impaired contractility^28^. Similarly, nitrosylation of the voltage-gated calcium channels (VGCCs) leads to their stimulation and renewal of calcium current inside the cell^29^.

All outlined scenarios could hardly be elucidated within the scope of a single study but these possibilities are worth investigating in the follow-up mechanistic studies of GSNO and, perhaps, several other thiols from our list.

Clinically wise, thiol compounds featured in this report demonstrate potential for use in a number of cardioprotective strategies. Currently, cardioplegia is widely used in cardiac surgery and heart transplantation. High potassium cardioplegic solution arrests ion-transport in cardiomyocytes and dramatically reduces oxygen/ATP demand to support the function of Na,K-ATPase, Ca-ATPases and myosin ATPase, primary energy consumers in cardiomyocytes. Protection of the heart through ischemic preconditioning has more complex and less clear molecular mechanism. It works reproducibly in animal studies but demonstrates much less convincing clinical benefits^30^. The use of (nitroso)thiols along with cardioplegia during cardiac surgery/transplantation may give additional protective value since these substances improve altered redox state of cardiomyocytes that is not a primary focus of the specialized salt solutions. At the same time, these substances may be incompatible with ischemic preconditioning since the latter uses ROS signaling, in particular^31^, whereas ROS are effectively discharged by thiols.

On the other hand, neither cardioplegia, nor direct ischemic preconditioning are useful methods for heart protection in patients with chronic ischemic heart disease. In these patients a periodic i.v. delivery of thiol/NO containing compounds is technically possible and worth be considered as a novel approach to myocardial protection and support. It appears more problematic to use such compounds in patients with myocardial infarction. Still, they could be promptly introduced in the affected region during recanalization of infarct related artery. We demonstrated that membrane penetrating substances et-GSH and tet-GSSG do not require preincubation to exert protective effect on cardiomyocytes. The use of such substances early at reperfusion may reduce an overall infarction size by protecting cardiac cells in the risk zone from damage under conditions of reoxygenation stress.

## Conclusion

Delivery of thiol-containing compounds in cardiomyocytes protects them from hypoxic damage and accelerates the recovery from ischemia-reperfusion stress. The basis of action of these substances includes regulatory glutathionylation of key ion transporters, direct quenching of ROS, protein nitrosylation and activation of NO-dependent signaling cascades. The net effect of such modifications is the stabilization of rhythmic Ca^2+^ transients and contractility of cardiomyocytes without Ca^2+^ overload. GSNO and other thiol-containing substances demonstrate low acute toxicity and could be further elucidated toward their cardiovascular application.

## Methods

### Rat Cardiomyocytes

Isolation and Manipulation of Rat Cardiomyocytes was extensively described in^32^. In brief, hearts were isolated from male Wistar rats anesthetized with ketamine (100 mg/kg). The protocol of experiments was approved by the Animal Ethics Committee of the Institute of Experimental Cardiology of the Russian Cardiology Research and Production Complex (# 3/15). All animal procedures were performed in accordance with the Institutional Guide for the Care and Use of Laboratory Animals. Rat heart was cannulated through aorta and perfused according to the method of Langendorf. Following washing steps heart was perfused in a recirculation mode for 30 min with 10 mL of 0.5 mg/mL CLS 2 collagenase (Worthington, 325 U/mg) solution in a Ca^2+^-free buffer. Digested heart was minced in fine pieces using scissors, and tissue was filtered through 200 micron Nylon mesh to separate isolated cardiomyocytes from undigested heart fragments. Live cardiomyocytes that settled down from suspension were gently resuspended in 10 mL of 6 mg/mL albumin solution, and 1 mM Ca^2+^ was reestablished in cell suspension by a stepwise addition of Ca^2+^ using 0.25 mM Ca^2+^ increments. Cardiomyocytes (Supplementary Fig. 5) were stored at 4 °C in Krebs-Henseleit solution supplemented with 1 mM Ca^2+^ and 6 mg/mL albumin up to 12 hours after isolation.

In single cardiomyocyte experiments, isolated cells were incubated in a test chamber in normoxic buffer (Tyrode solution aerated with carbogen 95% O_2_, 5% CO_2_ for 90 min) at 37 °C for 1–2 hours with continuous flashing the chamber over solution with carbogen. In order to reproduce hypoxic conditions, cardiomyocytes were placed in a Tyrode solution containing 1.2 mM Ca^2+^ and pre-aerated with a gas mixture of 95% N_2_, 5% CO_2_. Hypoxic gas mixture was continuously flashed over the hypoxic solution containing cardiomyocytes. Depending on experiment, the period of hypoxia lasted 2–15 min.

### Evaluation of Cell Viability

Viability of isolated cardiomyocytes was assessed using cell staining with Trypan Blue. To 10 μl of cardiomyocyte suspension (7.0 × 10^5^ cells/ml) 10 μl of 0.4% Trypan Blue solution was added. A drop of resulting mixture was placed under the microscope and microphotographs of 5–8 fields of vision in a bright field mode were taken using 10x objective. The total number of cells and the number of stained cells in each field of vision was counted and averaged. Cardiomyocyte viability is expressed as percent of live (unstained) cells in total cell suspension.

### Measurement of Ca^2+^ Transients in Isolated Rat Cardiomyocytes

We used procedure described in^32^. Briefly, cells were loaded with fluorescent Ca^2+^ indicator Fluo-4 (Invitrogen) at a final concentration 5–10 µM for 20 min and transferred in experimental chamber with glass coverslip as the bottom. Two platinum electrodes are attached at the opposite walls of the chamber and connected to Grass SD9 electrostimulator allowing pacing cardiomyocytes by rectangular impulses at 38 V and 1 Hz. The chamber is mounted on the stage of an inverted microscope in a thermostat set at 37 °C. Buffer flows through the chamber at 1–5 mL/min. Cardiomyocytes are monitored using x63 oil objective and high speed CCD camera. Fluorescence of Fluo-4 is excited using mercury lamp and an appropriate filter cube for FITC-based fluorophores. Fluorescent signal from the individual cardiomyocytes is recorded at 50–200 frames/sec and images are streamlined to a terabyte hard drive for further processing using AxioVision Physiology software (Zeiss). The following parameters of Ca^2+^ transients were digitized. 1) Time to peak was determined as time period in ms from the onset of Ca^2+^ transient to its peak. 2) Time to basal was measured as time period in ms from the peak of Ca^2+^ transient to its end at basal level. 3) Width at 30% height was measured as the duration of Ca^2+^ transient in ms at 30% height of this transient. All Ca^2+^ transient parameters were normalized by Ca^2+^ transient amplitude (fluorescence intensity, a.u.).

### Perfusion of isolated rat heart

The standard model of retrograde perfusion of isolated rat heart with carbogen-presaturated Krebs solution was used^32^. Constant perfusion pressure of 70 mm Hg was applied. Isovolumic pressure in the left ventricle was monitored using latex balloon placed in the left ventricle cavity. Perfusion protocol included several steps. After a 30-min perfusion with oxygenated Krebs solution, thiol-containing substance was added to this solution and the heart was perfused for additional 10 min. After this step the perfusion was stopped for 30 min. In the initial 10-min period of reperfusion thiol compound was present in oxygenated Krebs solution whereas the last 30 min of reperfusion were carried out with oxygenated Krebs solution only. Systolic and diastolic pressure and heart rate were recorded throughout the experiment. Since energy consumption by the myocardium is mainly determined by pressure development in the left ventricle and the frequency of contractions, to characterize heart output we calculated cardiac work index (CWI). CWI = (Ps − Pd) × HR, where Ps is systolic pressure, Pd is diastolic pressure, and HR is heart rate in Hz. Original traces of left ventricular pressure (LVP, in mmHg) of isolated rat hearts are given in the Supplementary Fig. 6.

### Cardioprotective substances

Nitrosoglutathione (GSNO, Sigma) is a glutathione derivative that causes an increase in intracellular glutathione levels, and affects both glutathionylation and nitrosylation of proteins. N-acetylcysteine (NAC, Sigma) is a cysteine derivative that penetrates the cell, acts as a reducing agent, and contributes to glutathione synthesis in cells. GSSG (AppliChem) is an oxidized glutathione; tetraethyl oxidized glutathione (tet-GSSG, was synthesized from GSSG by the authors) is cell-penetrating analogue of oxidized glutathione, and glutathione ethyl ester (et-GSH, Sigma) is a cell-penetrating analogue of reduced glutathione. To obtain working concentrations of these substances, we used 10 mM stock solutions of GSSG, GSNO, or tet-GSSG in 300 mM HEPES, pH 7.4; 10 mM GSNO in DMSO, and 100 mM NAC in 300 mM HEPES, pH 7.4. Depending on experiment, we either preincubated cardiomyocytes with these compounds for 20 min or omitted preincubation step. For toxicity evaluation the substances were added straight from the powder.

Glutathionylation of Na,K-ATPase α1 and α2 –subunit in samples was detected by immunoblotting as described lower. For immunoprecipitation of α1 subunit of Na,K-ATPase we used mouse monoclonal anti-Na,K-ATPase α1 antibody clone C464-6 (Upstate Millipore) and for immunoblotting we used dilution of antibodies 1:10000. For immunoprecipitation of α2 subunit of Na,K-ATPase, we used anti-Na,K-ATPase α2 subunit rabbit antibody AB9094-I (Merck Millipore) and for immunoblotting we used dilution of antibodies 1:4000.

### Immunoprecipitation and Immunoblotting

The levels of S-glutathionylation of Na,K-ATPase α1 and α2-subunit were estimated using immunoblotting after immunoprecipitation. Immunoprecipitation was performed on lysates of cardiomyocytes. Cells were lysed in cold RIPA buffer (1% Nonidet P-40, 1% sodium deoxycholate, 0.1%SDS, 150 mM NaCl, 0.2 mM phenylmethylsulfonyl fluoride, protease inhibitor cocktail 6 mg/mL, and 25 mM TrisHCl, pH 7.6) for 60 min at 4 °C. Anti-Na,K-ATPase α1 antibody clone C464-6 (Upstate Millipore) or anti-Na,K-ATPase α2 antibody rabbit antibody AB9094-I (Merck Millipore) were added to the cell lysate and the samples were incubated for 15 min at 4 °C and constant agitation. The resulting immune complex was added to a tube containing protein A agarose and incubated for 2 h at 4 °C and constant agitation. The samples were then centrifuged for 1 min at 15000 g and the supernatant was removed. The precipitate was washed with PBS for three times and then heated with 4x Laemmli buffer containing 8 M urea and 8% SDS at 80 °C for 5 min to elute the protein. Each sample was centrifuged and the supernatant was collected. Proteins of cell lysates were separated using 10% SDS-PAGE without mercaptoethanol or dithiothreitol and electrotransferred to a PVDF membrane. After the blocking procedure, mouse monoclonal anti-glutathione antibody (Chemicon Millipore, MAB5310, 1:1600) was added. Rabbit antiserum containing anti-Na,K-ATPase α2 antibody (Upstate Millipore, cat.# 07–674) (1:1000), α1 antibody clone C464-6 (Upstate Millipore) (1:10000) was applied to detect total amount of α2-subunit, α1-subuint followed by horseradish peroxidase-conjugated secondary antibodies. Membrane was developed for ECL using a commercial kit SuperSignal West Femto Maximum Sensitivity Substrate (Thermo Scientific) and chemiluminescence was detected using Bio-Rad ChemiDoc MP instrument. Densitometric analysis was performed by Image Lab (Bio-Rad) software and results were presented as the ratio of glutathionylated α2, α1 subunits to total α2 subunit, α1 subunit band intensity ((GSS-α2)/total α2), ((GSS-α1)/total α1, correspondingly. The ratio (GSS-α2)/total α2, (GSS-α1)/total α1 in control was taken as 1.

### Statistical analysis

Values are shown as means and standard deviations (SD). Data are presented as means of at least three independent experiments ± SD. For two groups comparision student’s t test was applied, and the difference was considered significant at p < 0.05. The comparison of data with multiple groups was performed using one-way ANOVA with post hoc testing using the paired samples Student’s t-test with Bonferroni correction (detailed information are presented in the figure legends). Statistical analysis was performed using STATISTICA 8.0 (StatSoft, Inc.).

## Supplementary information

Supplementary materials
