# Supplementary material for: Glutathione-related substances maintain cardiomyocyte contractile function in hypoxic conditions
Source: Sci Rep. 2019 Mar 19;9:4872. doi: 10.1038/s41598-019-41266-2 (PMC6425009; doi:10.1038/s41598-019-41266-2)
Supplement: Supplementary file 1 — Supplementary materials [file 41598_2019_41266_MOESM1_ESM.pdf]

Supplementary materials

Glutathione-related substances maintain cardiomyocyte contractile function in hypoxic conditions

Yuri M. Poluektov [1,2,#], Irina Yu. Petrushanko[1,#], Nidas A. Undrovinas[3], Valentina A. Lakunina[1] Asker Y. Khapchaev[3], Valery I. Kapelko[3], Alexander A. Abramov[3], Vladimir L. Lakomkin[3], Mikhail S. Novikov[4], Vladimir P. Shirinsky[3], Vladimir A. Mitkevich[1] and Alexander A. Makarov[1]*

[1]Engelhardt Institute of Molecular Biology, Russian Academy of Sciences, Vavilov St. 32, 119991 Moscow, Russia

[2]I.M. Sechenov First Moscow State Medical University, Ministry of Healthcare of the Russian Federation, Trubetskaya St. 8/2, 119991, Moscow, Russia

[3]National Medical Research Center for Cardiology, Ministry of Healthcare of the Russian Federation, 3rd Cherepkovskaya St. 15a, Moscow 121552, Russia

[4]Department of Pharmaceutical & Toxicological Chemistry, Volgograd State Medical University, Pavshikh Bortsov Sq., 1, Volgograd 400131, Russia

[#]These authors contributed equally to this work
*To whom correspondence should be addressed: Dr. Alexander A. Makarov, Engelhardt Institute of Molecular Biology, RAS, Vavilov St. 32, 119991 Moscow, Russia, Phone: +7 499 1354095, Fax: +7 499 1351405, E-mail: aamakarov@eimb.ru

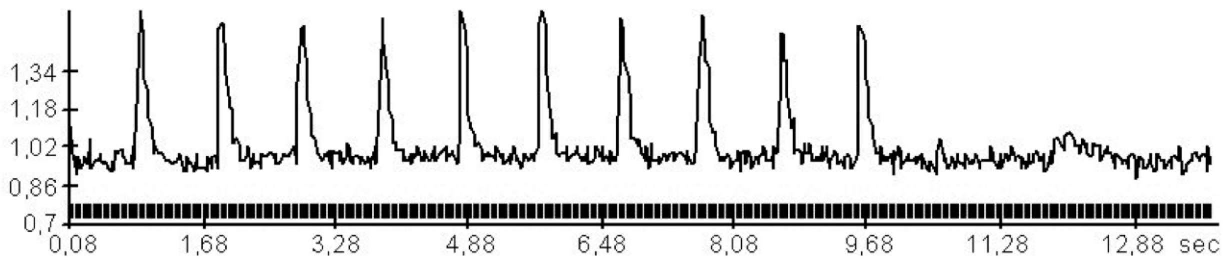

Supplementary Figure 1. Changes in the concentration of free intracellular Ca^{2+} in isolated rat cardiomyocytes during electric stimulation (1Hz) under hypoxic conditions (buffer saturated with 95% N2 and 5% CO_2) in the presence of et-GSH. 10 sec fragments of the record in the presence of 1 mM GSH, at 5 min of hypoxia is given. The ordinate shows the fluorescence intensity of Fluo-4 (arb. units).

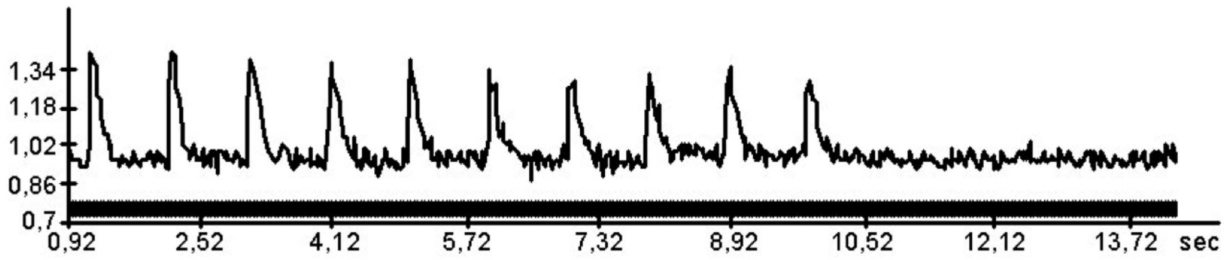

Supplementary Figure 2. Changes in the concentration of free intracellular Ca^{2+} in isolated rat cardiomyocytes during electric stimulation (1Hz) under hypoxic conditions (buffer saturated with 95% N2 and 5% CO_2) in the presence of NAC. 10 sec fragments of the record in the presence of 20 mM NAC, at 5 min of hypoxia is given. The ordinate shows the fluorescence intensity of Fluo-4 (arb. units).

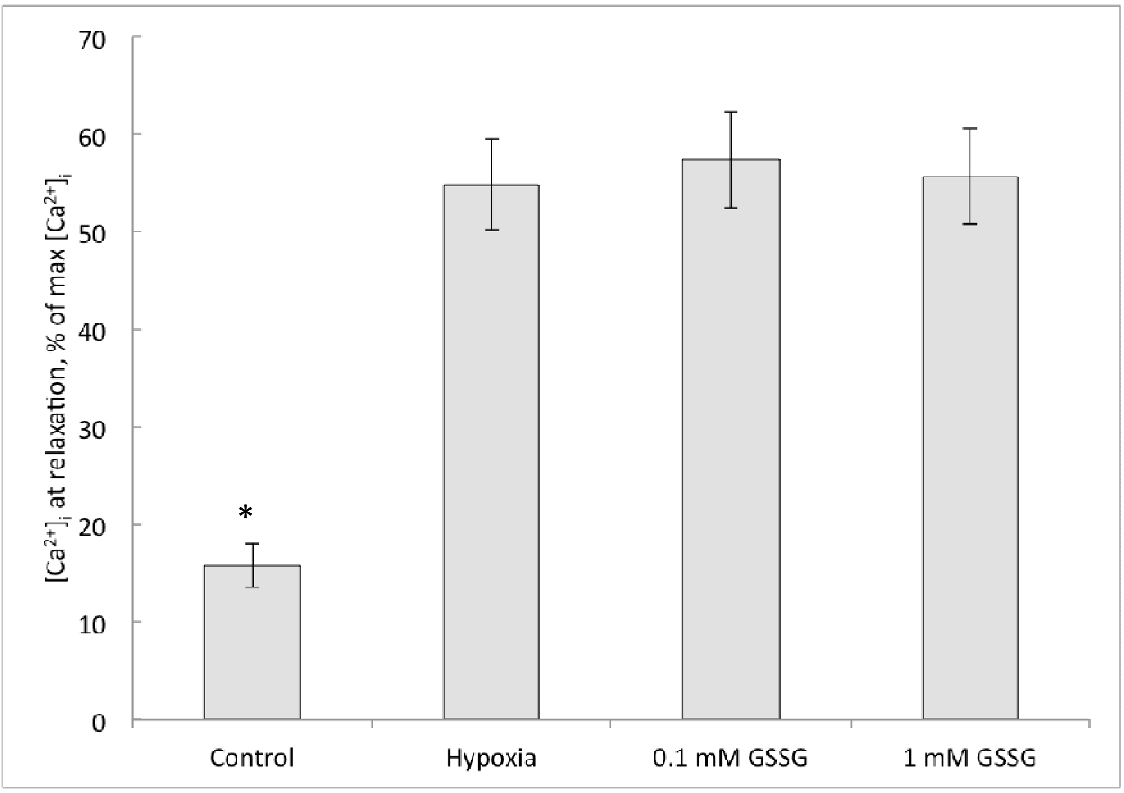

Supplementary Figure 3. Relative levels of basal Ca^{2+} in rat cardiomyocytes in control, hypoxic conditions and in hypoxic conditions in the presence of 0.5mM and 1mM GSSG without preincubation with this substance. The measurements were carried out at 2 min for control normoxia and hypoxia, and at 5 min for all other exposures. Average ratio ($[Ca^{2+}]$i at relaxation / $[Ca^{2+}]$i at peak) x 100% is presented for each condition. For all Ca^{2+} transients $[Ca^{2+}]$i at relaxation is determined at time point after Ca^{2+} peak in control normoxic cardiomyocytes when fluorescent signal reaches 20% of the peak value (on average at 250 msec). n = 3-9. Mean value ± S.D. Statistical analysis was performed using one-way ANOVA with post hoc testing (using the paired samples Student's t-test with Bonferroni correction); after a Bonferroni correction, p-value < 0.01 was considered as statistically significant. *- p <0.01 for all conditions (Hypoxia, 0.1 mM GSSG, 1 mM GSSG) vs Control.

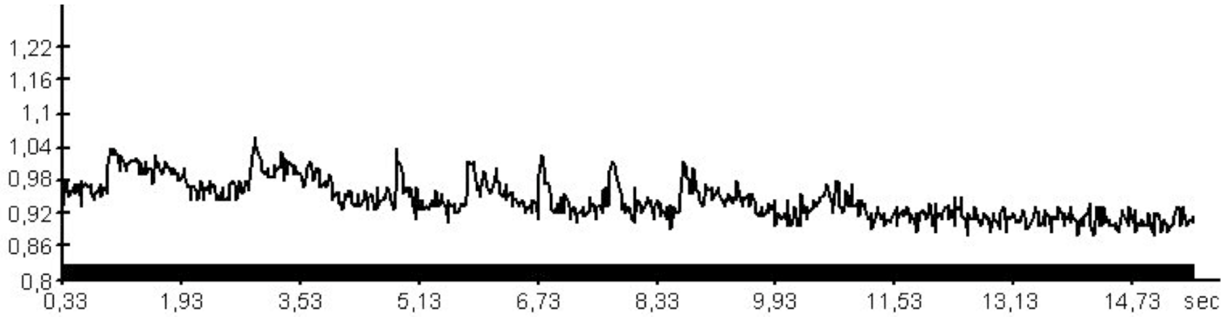

Supplementary Figure 4. Changes in the concentration of free intracellular Ca^{2+} in isolated rat cardiomyocytes during electric stimulation (1Hz) under hypoxic conditions (buffer saturated with 95% N_2 and 5% CO_2) in the presence of GSSG without preincubation. 10 sec fragments of the record in the presence of 1 mM GSSG, at 5 min of hypoxia is given. The ordinate shows the fluorescence intensity of Fluo-4 (arb. units).

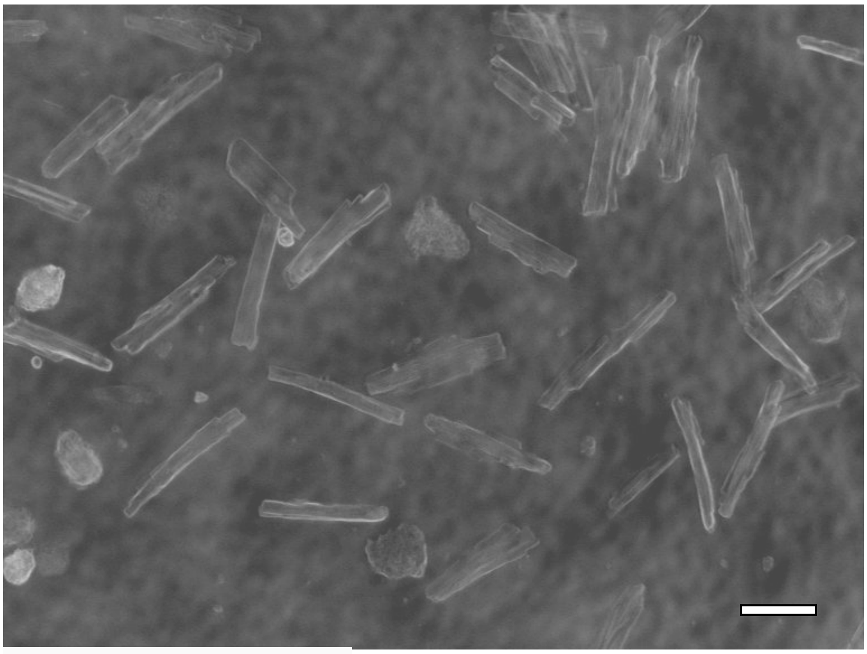

Supplementary Figure 5. Freshly isolated adult rat cardiomyocytes in normoxic buffer. Phase contrast. Bar, 50µm.

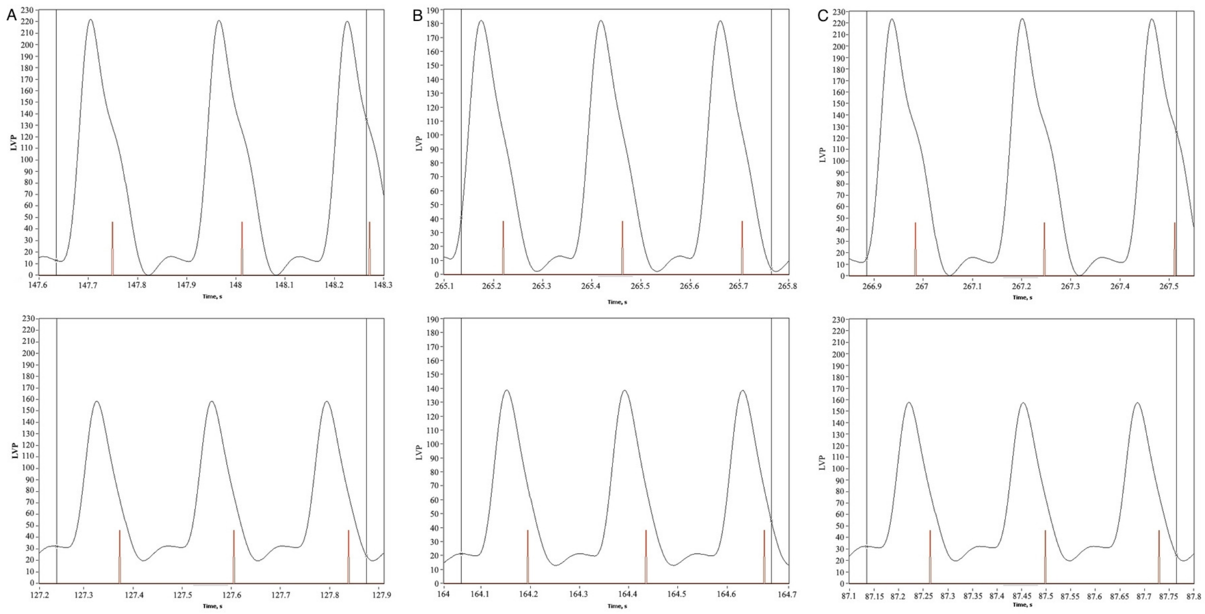

Supplementary Figure 6. Original traces of left ventricular pressure (LVP, in mmHg) of isolated rat hearts: (A) control, (B) 0.05 µM GSNO, (C) 25 µM GSNO. Each panel with pressure traces is the screenshot of processing program window with the equal time intervals – 700 ms. Upper panels represent initial LVP traces before ischemia. Bottom panels represent LVP traces after 30 min ischemia and 40 min reperfusion. Red spikes in bottom of all panels are marks of heart beats during processing of raw pressure curves.

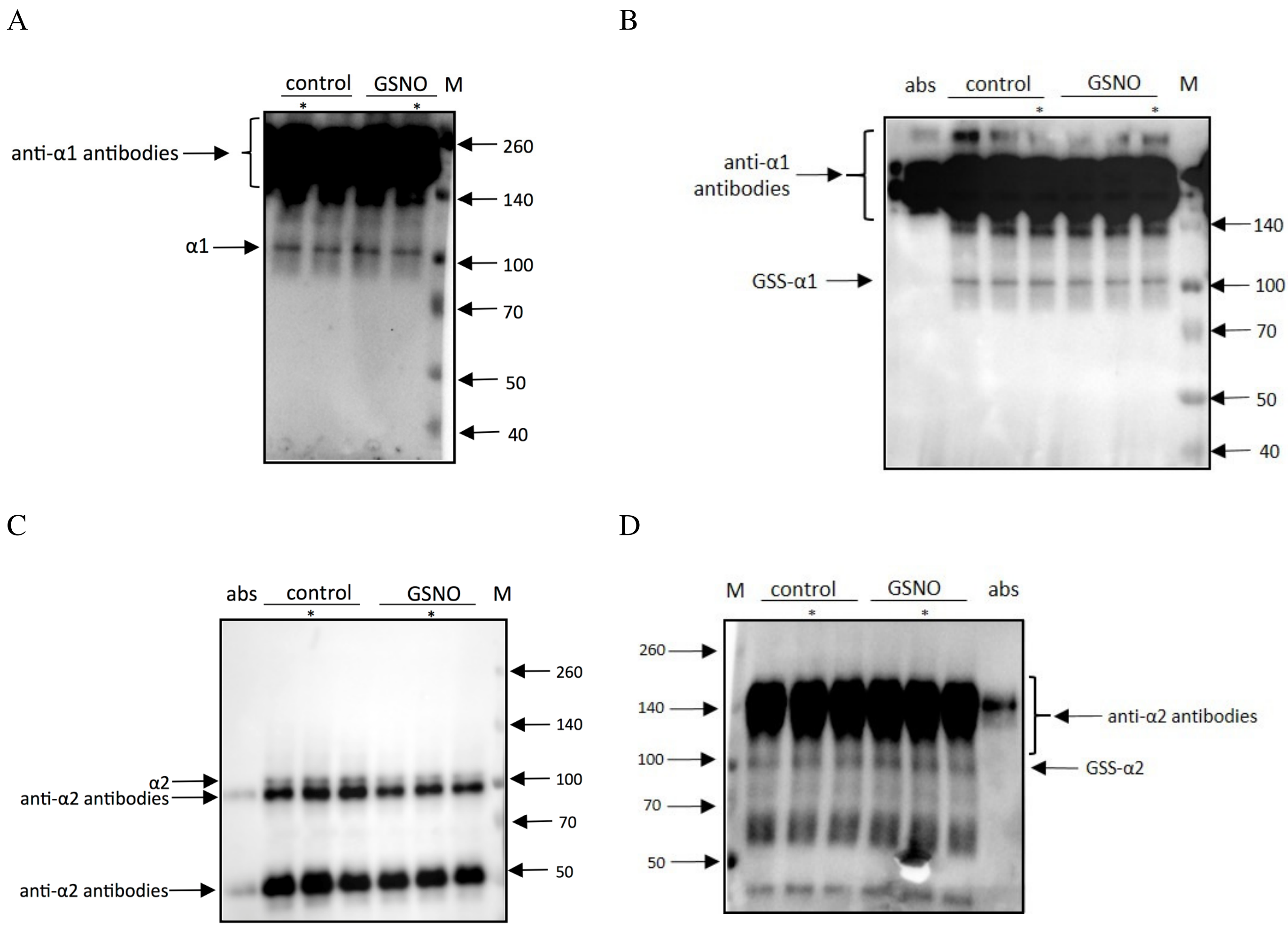

Supplementary Figure 7. Full length Western blots corresponding to Fig.6. A, B – Western blot with immunoprecipitated α1 subunit, detected with anti-alpha1 (A) and anti-glutathione (B) antibodies. C,D - Western blot with immunoprecipitated α2 subunit, detected with anti-alpha2 (C) and anti-glutathione (D) antibodies. K – control cells; GSNO – cells, treated with 0.5mM GSNO; abs – control immunoprecipitation performed with antibodies used in the assay, but without cell lysates to follow the electrophoretic mobility of antibodies; M – the marker of molecular weights. Lanes that contain bands presented in the paper, are marked with *.
